# Treatment of elderly patients with refractory/relapsed multiple myeloma: oral drugs adherence and the COVID-19 outbreak

**DOI:** 10.18632/oncotarget.27819

**Published:** 2020-11-24

**Authors:** Flávia Dias Xavier, Fernando Sergio Blumm Ferreira, Rodrigo Martins Abreu

**Affiliations:** ^1^Department of Hematology, Hospital Universitario de Brasilia-UNB/Ebserh, Brasilia, DF, Brazil; ^2^Hospital Sirio Libanes, Centro de Oncologia, Unidade Brasilia, Brasilia, DF, Brazil; ^3^Medical Affairs, Takeda Distribuidora Ltda., São Paulo, SP, Brazil

**Keywords:** healthcare, multiple myeloma, adherence, elderly, refractory/relapsed

## Abstract

Once the treatment of refractory/relapsed multiple myeloma in the elderly is greatly influenced by the adherence of patients and family members, clinicians should be aware of patients’ behavior and lifestyle, as it may influence the individual treatment plan for each patient. Furthermore, treatment with oral chemotherapy is of special value during the COVID-19 outbreak. Multidisciplinary healthcare involvement is crucial in the management of polypharmacy, adverse events and dose adjustment due to comorbidities and natural loss of renal function with age. Oral drugs simplify intake, reduce hospital visits, and improve autonomy and quality of life. However, although oral drugs have advantages, they also transfer control and responsibility from the healthcare professional to the patient, who must be able to understand and follow the directions given. Therefore, patient education and communication with healthcare professionals are critical for adherence.

## INTRODUCTION

Multiple myeloma (MM) is a treatable, although incurable, disease that represents 2% of malignant neoplasms and affects patients with a median age of 69 years old (two-thirds ≥ 65 years, one-third ≥ 75 years and 10% ≥ 85 years) [1, 2]. In Brazil, there are 29 million citizens aged > 60 years [3], and by 2030, the life expectancy will be 79 years [4, 5], with the number of elderly people increasing by 40% [3]. The incidence of MM follows the population trend. Between 2010 and 2030, an increase of 57% is expected among the general population, with an increase of 68% among people ≥ 65 years old [6].

**Figure 1:**
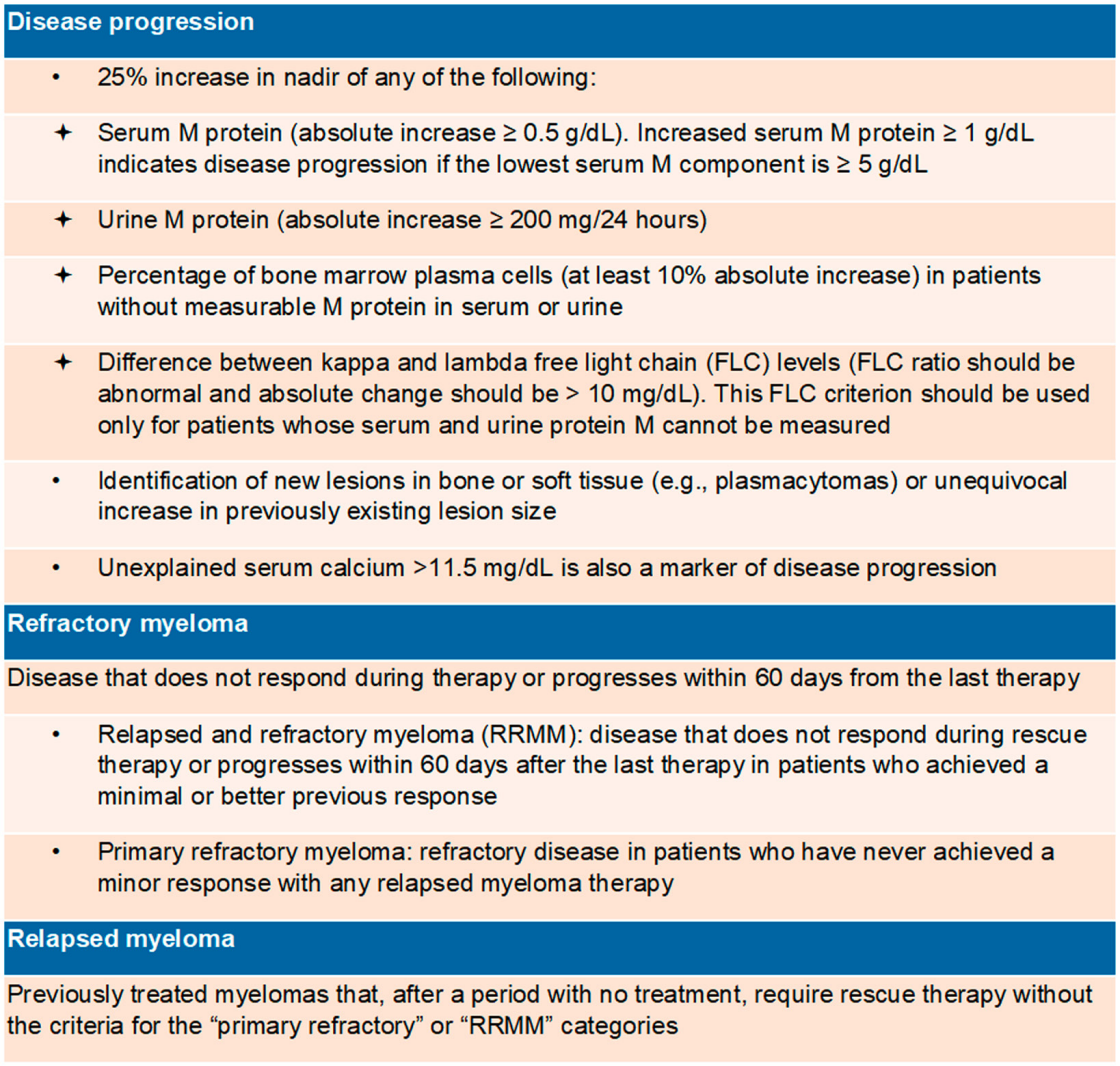
Definitions [11, 85].

The frequent relapses and continuous treatment requirements of MM (Figure 1), together with its prevalence in the elderly population, make the individualization of therapeutic choice essential, with a special focus on adherence to treatment, which may impact patients’ physiological and socioeconomic reality [7].

**Figure 2:**
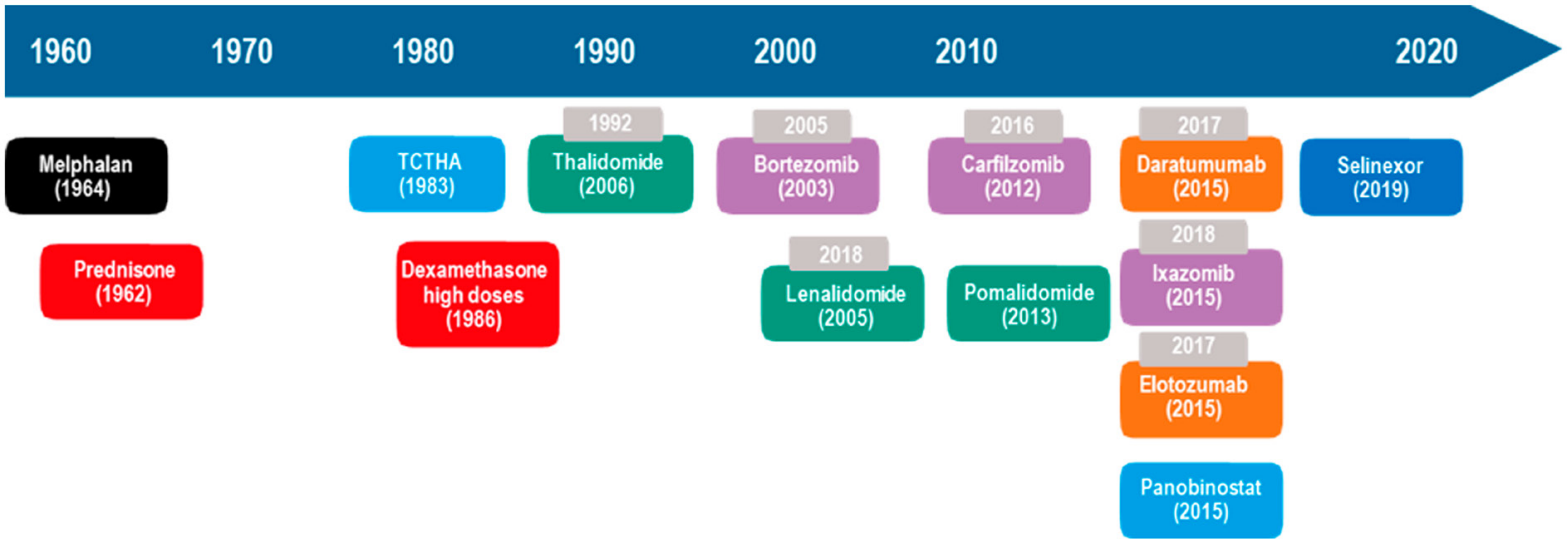
Evolution of MM treatment in the US. Gray = year of approval in Brazil. Black = alkylating agents. Red = corticosteroids. Green = immunomodulators. Purple = proteasome inhibitors. Orange = monoclonal antibodies. Blue = histone deacetylase inhibitor. auto-SCT = autologous hematopoietic stem cell transplantation. Dark blue = selective inhibitor of the nuclear export protein exportin 1 (XPO1). Oral or intravenous drugs: melphalan and dexamethasone. Oral drugs only: prednisone, thalidomide, lenalidomide, pomalidomide, ixazomib, panabinostat and selinexor. Intravenous only: Carfilzomib and elotozumab. Intravenous or subcutaneous: bortezomibe and daratumumab.

The introduction of new drugs for MM treatment (Figure 2) resulted in an increase in 5-year survival from 29% (1990) to 54% (2015) [1] and an increase in survival time from 3.2 (2001–2005) to 5 years (2006–2010) in patients > 65 years old [8], but there was no improvement in survival at > 80 years old [9, 10]. In addition, out of the ten antimyeloma drugs that have emerged in the last 21 years, seven are oral (Figure 2). In clinical studies, combinations of daratumumab, lenalidomide, and dexamethasone (DRd); carfilzomib, lenalidomide, and dexamethasone (KRd); elotuzumab, lenalidomide, and dexamethasone (ERd); ixazomib, lenalidomide, and dexamethasone (IRd); daratumumab, bortezomib, and dexamethasone (DVd); pomalidomide, bortezomib, and dexamethasone (PVd); and panobinostat, bortezomib, and dexamethasone (Pan-Vd) are more efficacious than the combination of lenalidomide and dexamethasone (Rd) or bortezomib and dexamethasone (Vd) in the treatment of relapsed and/or refractory MM (RRMM) (Table 1), resulting in better responses and increased progression-free survival (PFS) and overall survival (OS) [11–31].

**Table 1:** First to third lines of rescue treatment

Studies	POLLUX [11, 12, 17, 29]	ASPIRE [26–30]	ELOQUENT-2 [13, 14, 31]	TOURMALINE-MM1 [15]	CASTOR [11, 16–18]	ENDEAVOR [19, 20, 30]	OPTIMISMM [21]	PANORAMA1 [22]
Experimental versus	DRd versus Rd	KRd versus Rd	ERd versus Rd	IRd versus Rd	DVd versus Vd	Kd versus Vd	PVd versus Vd	Pan-Vd versus Vd
Control								
*N* (patients)	286 versus 283	396 versus 396	321 versus 325	360 versus 362	251 versus 247	464 versus 465	281 versus 278	387 versus 381
OR (%)	**93 versus 76(s)**	**87 versus 67(s)**	**79 versus 66(s)**	**78 versus 72(s)**	**83 versus 63(s)**	**77 versus 63(s)**	82.2 versus 50	60.7 versus 54.6
≥ VGPR (%)	**79 versus 48(s)**	**70 versus 40(s)**	35 versus 29	**48 versus 39(s)**	**59 versus 29(s)**	**54 versus 29(s)**	52.7 versus 18.3	27.6 versus 15.7
≥ CR (%)	**51 versus 21(s)**	**32 versus 9(s)**	5 versus 9	**12 versus 7(s)**	**19 versus 9(s)**	**13 versus 6(s)**	15.7 versus 3.9	11 versus 6
MRD neg (< 10–5) (%)	26 versus 6(s)	NR	NR	NR	7 versus 2(s)	NR	NR	NR
Median PFS (months)	**NA versus 18(s)**	**26 versus 17(s)**	**19 versus 15(s)**	**21 versus 15(s)**	**17 versus 7(s)**	**18 versus 9(s)**	**11.2 versus 7.1 (s)**	**11 versus 8(s)**
PFS (%)	**24 months:** **68 versus 41(s)**	**18 months:** **65 versus 47(s)**	**48 months:** **21 versus 14(s)** **24 months:** **41 versus 27(s)**	NR	**18 months:** **48 versus 8(s)**	**18 months:** **49 versus 24(s)**	NR	24 months: 21 versus 8
Reduction of DP risk/death (%)	63	31	29	25	69	NR	NR	NR
Duration of response (%)	NA versus 17	29 versus 21	21 versus 17(s)	21 versus 15	NA versus 8	21 versus 10	8.8 versus 4.9	13 versus 11
Median OS (months)	NA versus 20(ns)	**48 versus 40(s)**	**48 versus 40(s)**	NA versus NA	NR	**48 versus 40(s)**	NR	33.6 versus 30.4
OS (%)	12 months: 92 versus 87(ns)	**67.1 months:** **33 versus 25(s)**	48 months: 50 versus 43 (nr)	NR	NR	**37 months:** **59 versus 55(s)**	NR	NR
Median for the 1st response (months)	1 versus 1.3	1.6 versus 2.3	2.8 versus 2.8	1.1 versus 1.9	0.9 versus 1.6	1.1 versus 1.1	**0.9 versus 1.4 (s)**	1.5 versus 2
Median follow -up (months)	25.4	67.1	48	23	19.4	37.5	15.9	6.5

(ns) = not significant. (s) = significant (*p* < 0.05) (bold). NR = not reported. NA = not available. Abbreviations: significant (*p* < 0.05), s; not reported, NR; not reached, NA; progression-free survival, PFS; overall survival, OS; overall response, OR; very good partial response, VGPR; complete response, CR; minimal residual disease, MRD; versus.

In this context, the objective of this perspective paper is to review MM management with oral therapies, focusing on adherence in elderly patients with RRMM along with clinical experiences and perspectives in Brazil.

### Adherence to oral drugs

Low adherence may lead to drug resistance; poor response to treatment; disease progression; increased medical appointments, laboratory tests, hospitalizations and death; and increased health costs [32]. In the US, lack of adherence is the largest preventable factor in healthcare cost, accounting for over $200 billion/year [33, 34].

**Figure 3:**
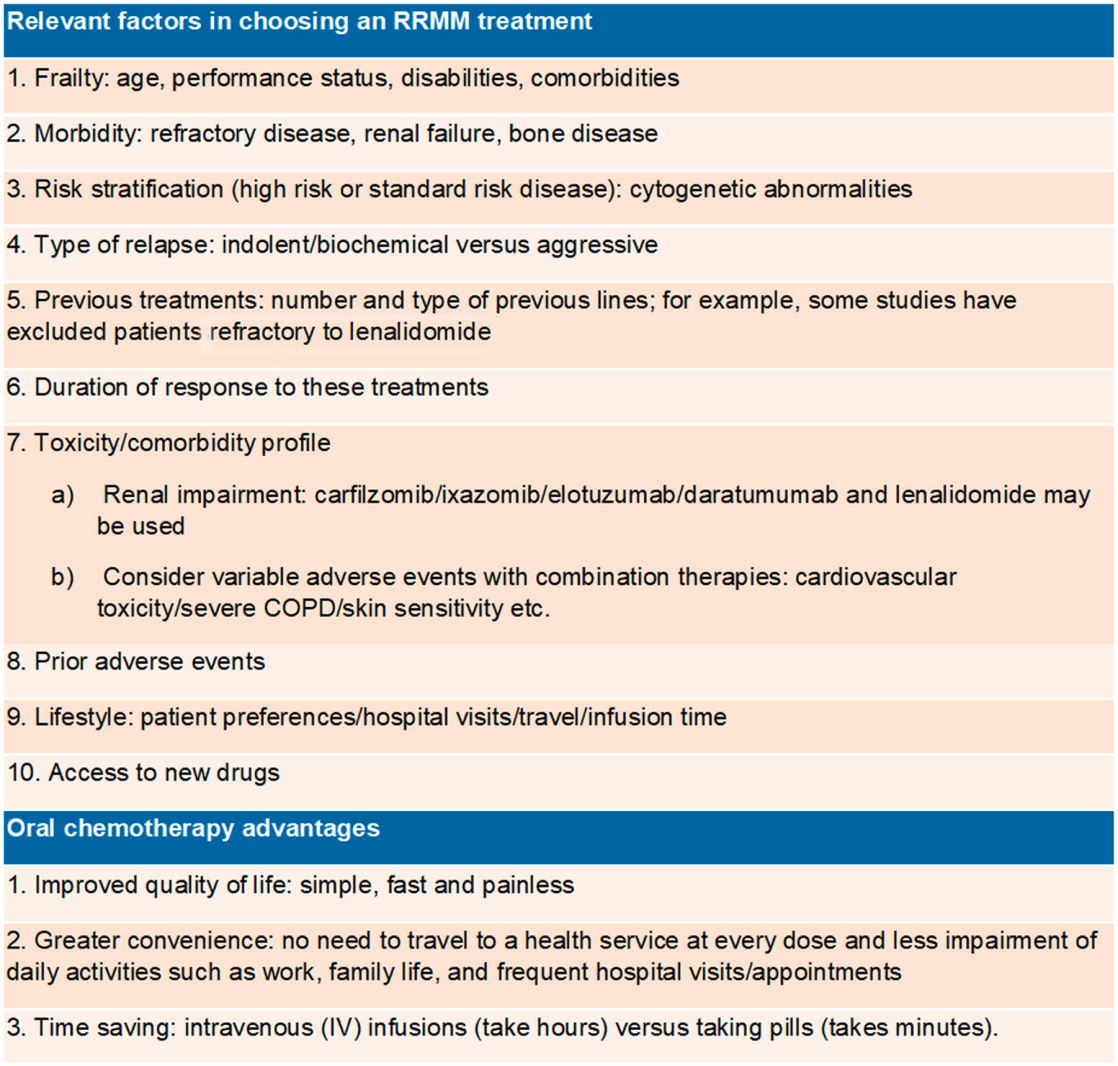
Relevant factors in the therapeutic choice and oral chemotherapy [7].

**Figure 4:**
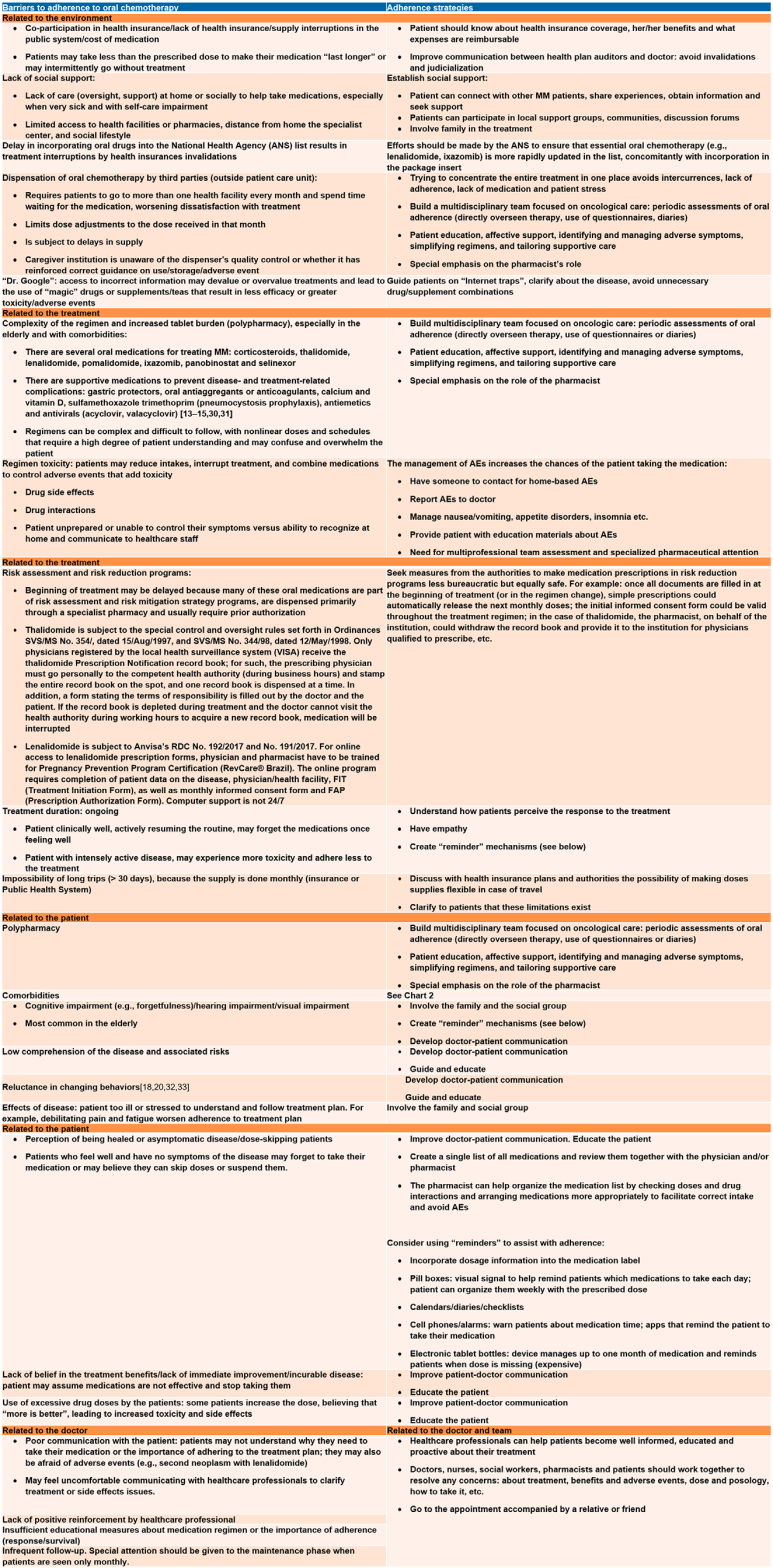
Barriers to adherence and strategies for adherence to oral chemotherapy [65, 66]. Some items in the table are based on our experience.

Of the seven available myeloma oral medications, four are available in Brazil (melphalan, thalidomide, lenalidomide and ixazomib). However, in the public health system, of which 72,1% of the population depends exclusively [3], only thalidomide and melphalan are available. Thalidomide is usually combined with cyclophosphamide and dexamethasone as induction for transplant-eligible myeloma patients, while melphalan is combined with prednisone with or without thalidomide as first-line treatment of transplant-ineligible patients. Oral drugs have advantages (Figure 3), but they shift the focus of control and responsibility from the healthcare professional to the patient, who must be able to understand and follow all the directions given by the drug’s prescriber. Barriers to adherence are higher for older patients (Figure 4); therefore, we believe that physicians should educate, instruct, and monitor their patients as well as encourage them to adhere to treatment.

We understand that multidisciplinary involvement, especially of pharmacists, is essential in the management of polypharmacy (optimizing efficacy and reducing adverse events (AEs) and drug interactions), as is the cooperation of the family or other supportive social group—especially in the case of elderly patients whose adherence may be compromised by inability to follow the treatment plan, by forgetting to take medications, and by a lack of assistance from third parties in administering medications or accompanying patients to medical appointments, as well as by AEs associated with medication use. In our practice, AEs can also influence medical staff to reduce the dose or withhold the drug. Additionally, treatment is effective only if followed and is followed only if well tolerated.

### How to choose the best salvage treatment for elderly patients: balancing efficiency and tolerance to promote adherence

Frailty is an accumulated decline in many physiological systems, resulting in reduced resistance to stressors such as cancer and its treatment in a manner that is unpredictable by the Eastern Cooperative Oncology Group performance status (ECOG); this decline impacts adherence [35].

The International Myeloma Working Group (IMWG) [35] has created a frailty score that predicts mortality and the risk of toxicity in the elderly (Table 2). The IMWG recommends that fit patients receive triple therapy at a full dose or higher (autologous hematopoietic stem cell transplantation (auto-SCT)); that intermediate-fit patients receive less intense double or triple treatments (reduced dose) [36]; and that frail patients receive double reduced-dose or even palliative/supportive therapy due to the benefits of low toxicity for the survival of very fragile patients.

**Table 2:** Frailty score [35, 82–84]

Factors	Points
≤ 75 years	0
76–80 years	1
> 80 years	2
***Activities of Daily Living (ADL)*^#^**	
> 4	0
≤ 4	1
***Instrumental Activities of Daily Living (IADL)*^##^**	
> 5	0
≤ 5	1
***Charlson Comorbidity Index (CCI)*^###^**	
≤ 1	0
≥ 2	1
**Classification:**	
Fit	0
Intermediate fitness	1
Frail	2 to 5

***N* = 869 (%) **	**SG 3 years (%)**	**SLP 3 years (%)**	**Nonhematological AEs, grade ≥ 3 (%)**	**Treatment discontinuity**	**Deaths**
39	84(s)	48(s)	22(s)	17%(s)	10%
31	76(s)	41(s)	26(s)	21%(s)	14%
30	57(s)	33(s)	34(s)	31%(s)	27%

(s) = significant (*p* < 0.05) (bold). ^#^Score (0 to 6) - 1 point: Bathing, dressing, using the toilet, transfer, continence and eating. ^##^Score (0 to 8) - 1 point: phoning, shopping, preparing food, taking care of the house, doing laundry, transportation, taking care of own medications, taking care of finances. ^###^Score (0 to 37) - 1 point: heart attack, heart failure, peripheral arterial disease, stroke, dementia, chronic obstructive pulmonary disease (COPD), collagenosis, peptic ulcer, mild liver disease, uninjured target-organ diabetes. 2 points: hemiplegia, at least moderate renal failure, injured target-organ diabetes, nonmetastatic cancer, leukemia, lymphoma. 3 points: at least moderate liver disease. 6 points: metastatic cancer, AIDS. Available online: http://www.myelomafrailtyscorecalculator.net/Geriatric.aspx.

In the eight Phase III studies (Table 1 and Supplementary Table 1), which compared triple versus double therapy or carfilzomib and dexamethasone (Kd) versus Vd in RRMM, patients ≥ 65 years represented half of the population (42–58%), and up to one-fifth of all participants were ≥ 75 years old (9–21%); however, the included patients had ECOG ≤ 2 (> 90%: 0–1) and had no significant comorbidities. In most patients (65–74 or ≥ 75 years), there was a significant gain of PFS in the experimental arm (triple rescue or Kd) in relation to the control arm (double therapy or Vd). Triple rescue or Kd therapy may be considered in elderly patients with ECOG scores of 0–1.

### Comorbidities and toxicities

The IMWG recommends the use of the Chronic Kidney Disease Epidemiology Collaboration equation (preferred) or the Modification of Diet in Renal Disease Study Group equation to assess renal function [37]. In addition to light chain associated renal impairment or hypercalcemia by MM, there is a natural loss of renal function with age, and approximately half of adults > 70 years old have creatinine clearance (CrCl) < 60 mL/min [38, 39]. Elderly population have a higher incidence of comorbidities with an impact on renal function, such as diabetes and hypertension, as well as higher usage rates of nephrotoxic drugs such as nonsteroidal anti-inflammatory drugs [38, 39].

Patients with CrCl < 30 mL/min are underrepresented (6% ENDEAVOR and < 1% TOURMALINE-MM1) in clinical studies. CrCl of 30–50 or 60 mL/min represented a 10 to 30% of the study population. PFS increased in patients with CrCl < 60 mL/min taking DRd, DVd and Kd [12–20, 23, 25–31].

In general, all salvage treatments can be tolerated, as long as the dose is adjusted for renal function (lenalidomide, pomalidomide and ixazomib) to prevent AEs [24, 40–43]. Bortezomib is a tolerable drug in the context of renal failure. Although carfilzomib does not require dose adjustment, there are few data from individuals with CrCl < 15 mL/min [37, 44, 45]. Panobinostat and selinexor do not need dosage adjustments for renal failure, although their dialyzability is unknown [46, 47].

In several studies, DVd was associated with higher neutropenia and thrombocytopenia rates than Vd was (Table 3) [12, 16–18]. Kd was associated with a slight increase in anemia and thrombocytopenia compared to Vd (based ENDEAVOR study) [19, 20, 30]. All triple therapies induced G3/4 anemia at a rate of 14–20%, which did not differ from the rate in the control group (Vd or Rd) [12–23, 25–31]. The DRd [12, 16, 17, 23, 25] and PVd [21] protocols resulted in G3/4 neutropenia at a rate of more than 50%, while Vd resulted in a rate of 4–9% [12, 16–18, 21–23, 25]. The Pan-Vd and DVd protocols were the ones that most resulted in G3/4 thrombocytopenia (68% and 45%) [11, 16–18, 22], while Kd resulted in < 10% thrombocytopenia (Table 3) [19, 20, 30]. Thalidomide has limited hematological toxicity, but bortezomib, carfilzomib, lenalidomide and alkylating agents often cause thrombocytopenia [48]. Weekly bortezomib reduced the frequency of AEs compared to biweekly bortezomib and is preferred for fragile patients [48–50].

**Table 3:** Hematological toxicity and peripheral neuropathy with rescue protocols

Adverse events (AEs)	Anemia		Neutropenia		Thrombocytopenia		Peripheral neuropathy		Second primary neoplasm
Toxicity	All	G3/4	All	G3/4	All	G3/4	G0-5 (%)	≥ G3 (%)	—
POLLUX daratumumab arm* [11, 12, 23, 25]	37	16	**61**	**54**	29	14	NR	NR	5.7
ASPIRE carfilzomib arm^#^ [26–30]	43	19	**40**	31	29	**17**	19	3	NR
ELOQUENT 2 elotuzumab arm^&^ [13, 14, 31]	97	20	83	**36**	**84**	21	NR	NR	*
TOURMALINE-MM 1 ixazomib arm^#^ [15]	29	**9**	33	23	**31**	**19**	27	2	5
CASTOR daratumumab arm* [11, 16–18]	26	14	**18**	**13**	**59**	**45**	**50**	**5**	4.1
ENDEAVOR carfilzomib arm^##^ [19, 20, 30]	**40**	15	NR	NR	21	9	10	1	NR
OPTIMISMM pomalidomide arm [21]	28	13.7	46.8	82.1	36.7	27.3	47.8	8.3	3
PANORAMA1 panobinostat arm [22]	62	18	75	35	98	68	61	18	NR
Lenalidomide, dexamethasone (POLLUX) [11, 12, 17, 25]	39*	21	45	40	31	16	17–22	3	3.6–5.7
Lenalidomide, dexamethasone (ASPIRE) [26–30]	40^#^	18	35	28	24	13			
Lenalidomide, dexamethasone (ELOQUENT-2) [13, 14, 31]	95^&^	21	89	45	78	21			
Lenalidomide, dexamethasone (TOURMALINE-MM1) [15]	27^##^	13	31	24	16	9			
Bortezomib, dexamethasone (Castor) [11, 16–18]	31^*^	16	9	4	44	33	**29–38**	**7**	1
Bortezomib, dexamethasone (ENDEAVOR) [19, 20, 30]	28^##^	11	NR	NR	17	9	**29**	**6**	NR
Bortezomib, dexamethasone (OPTIMISMM) [21]	27	14	11	9	38	29	**37**	**4**	1
Bortezomib, dexamethasone (PANORAMA1) [22]	52	19	48	8	84	31	**67**	**15**	

* All degrees (≥ 15%) and degree 3/4 (≥ 5%); ^#^(≥ 20%); ^&^(≥ 30%); ^##^≥ 10% and degree 3/4/5 ≥ 2%.

An ASPIRE subanalysis compared patients < 70 years to patients ≥ 70 years, and the frequencies of AEs ≥ grade 3, such as neutropenia, anemia, and thrombocytopenia, were increased in carfilzomib, lenalidomide, and dexamethasone patients aged ≥ 70 years (37%, 24% and 20%, respectively) compared to both the control group (23%; 21%; 15%) and KRd patients aged < 70 years old (28%; 16%; 11%) [51]. The use of granulocyte colony stimulating factor (GCSF) and erythropoietin can be considered for the management of neutropenia and anemia, respectively [50].

Peripheral neuropathy (PN) (Table 3) may be caused by comorbidities (e.g., diabetes), MM, or treatment with thalidomide and/or bortezomib [48, 50]; when caused by these medications, it can be cumulative and relates to the duration of exposure [52, 53]. In RRMM, it is essential to review the AEs of first-line regimens, since melphalan, prednisone and thalidomide can result in PN at a rate of up to 55% (10% G3/4) [48, 54, 55] while bortezomib, melphalan and prednisone (VMP) can result in up to 44% (14% G3/4) PN [56]. Subcutaneous weekly infusions of bortezomib significantly reduced PN without changing the endpoint [57, 58]. For patients with pre-existing PN or comorbidities that render them unable to tolerate PN, lenalidomide-based regimens are preferable. In such cases, it is necessary to combine proteasome inhibitors, preferably carfilzomib (< 3% PN at < 70 years or ≥ 70 years) [51] or ixazomib [15], with bortezomib. In current PN, gabapentin, pregabalin or duloxetine and opioids may improve symptoms and contribute to treatment adherence.

In an ASPIRE subgroup analysis comparing patients < 70 years to those ≥ 70 years, heart disease (9%) and ischemia (5%) of grade ≥ 3 were more frequent in patients ≥ 70 years (KRd) than in the control group (Rd) (2% and 1%) or in patients < 70 years (KRd) (2% and 3%) [51]. Discontinuation due to cardiovascular AEs was higher in patients aged ≥ 70 years than in younger patients (6.8% versus 1.4%) [51]. When choosing a treatment, clinicians should consider cardiovascular toxicities (congestive heart failure and myocardial ischemia), especially in elderly or frail patients or those with previous heart disease. In these cases, physicians may opt for a non-carfilzomib regimen, as carfilzomib maybe results in a congestive heart failure rate of up to 5%, with a death rate of < 1% [19, 26].

Patients with known asthma and chronic obstructive pulmonary disease (COPD), with forced expiratory volume < 50% or moderate or serious persistent asthma in the last two years, should be carefully evaluated before receiving daratumumab due to the risk of bronchospasm; therefore, any patient with a history of COPD should be evaluated for concurrent use of short- and long-term bronchodilators and inhaled corticosteroids. Patients with mild asthma should take inhaled bronchodilators for the first infusion [59]. Discontinuation due to infusion-related reactions is rare (< 1%) [59].

### Cytogenetic abnormalities and type of relapse

Patients with high-risk cytogenetics abnormalities (del17p, t [4, 14] and t [14, 16]) have worse outcomes than those with typical cytogenetics. T (4;14) occurs in 11% and 8% of patients aged 66–75 and > 75 years, respectively, while del17p is observed in 6% of both age groups [60].

Regarding cytogenetics abnormalities, consideration should be given to choosing treatments that improve or overcome the poor prognosis of the disease (Supplementary Table 2). According to IMWG guidelines, patients with cytogenetics abnormalities should be rescued with regimens that combine proteasome inhibitors and immunomodulatory drugs [61]. In TOURMALINE-MM1, IRd was able to overcome the negative impact of high-risk cytogenetic abnormalities (PFS 21.4 months IRd versus 9.7 months in Rd, *p* = 0.021) (Supplementary Table 2) [15]. Therefore, IRd may be an oral option for elderly patients.

In the case of biochemical relapse, especially during maintenance therapy, increasing the current drug dose and subsequently adding another agent is a recommended strategy [45]. In aggressive relapses with fast-growing tumors, new bone lesions, extramedullary disease, cytogenetics abnormalities, high lactate dehydrogenase levels and plasma cell leukemia, more aggressive therapy should be instituted with three- or four-drug regimens [7], while frail or elderly patients may be candidates for oral and well-tolerated regimens. In general, the regimens in Table 1 may be suitable for both situations.

### Response duration and previous treatment

Elderly MM patients have a persistently worse prognosis than younger patients, which may be related to the higher frequency of treatment discontinuation and AEs [35]. In addition, elderly and frail patients are included in clinical trials less often than younger, healthier patients and therefore, may have less access to new drugs. There are also comorbidities and drug interactions that can complicate treatment, limit physical condition and impair adherence.

Repeating the same treatment is an option for patients with a lasting response, i.e., > 20–24 months after the first induction or > 9–12 months after rescue therapy [45]. Lower response duration should be treated with an alternative regimen. Patients who relapse < 12 months after the first line or while undergoing treatment (refractory disease) should be treated as high risk regardless of their fluorescence *in situ* hybridization results [62].

In general, a second course of auto-SCT is not recommended in patients who relapse in < 12–18 months (without maintenance) and < 36 months (with lenalidomide maintenance), as the second course of auto-SCT is likely to have an even smaller benefit than the first in terms of PFS [63].

On ERd, patients with a mean diagnosis time ≥ 3.5 years had an advantage in PFS compared to those taking Rd (HR 0.59, CI: 0.45–0.78); the greatest benefit was observed in the subset with mean diagnosis time ≥ 3.5 years and one previous line (30.4 versus 19.4 months, *p* = 0.0224), with a 44% reduction in the risk of progression or death [14].

DRd versus Rd PFS benefits were maintained at prior treatment exposure time since last therapy > 12 months (not applicable (NA) versus 25 months, *p* < 0.0001), ≤ 12 months (29 versus 10 months, *p* < 0.0001), > 6 months (NA versus 21 months, *p* < 0.0001) and ≤ 6 months (NA versus 10 months, *p* = 0.0002) [25].

The benefit in PFS with DVd versus Vd was maintained regardless of the time since last therapy (≤ 12, > 12, ≤ 6 or > 6 months: 10 versus 5 months, NA versus 9.4 months, 10 versus 5 months and 20 versus 9 months, respectively) [18].

**Figure 5:**
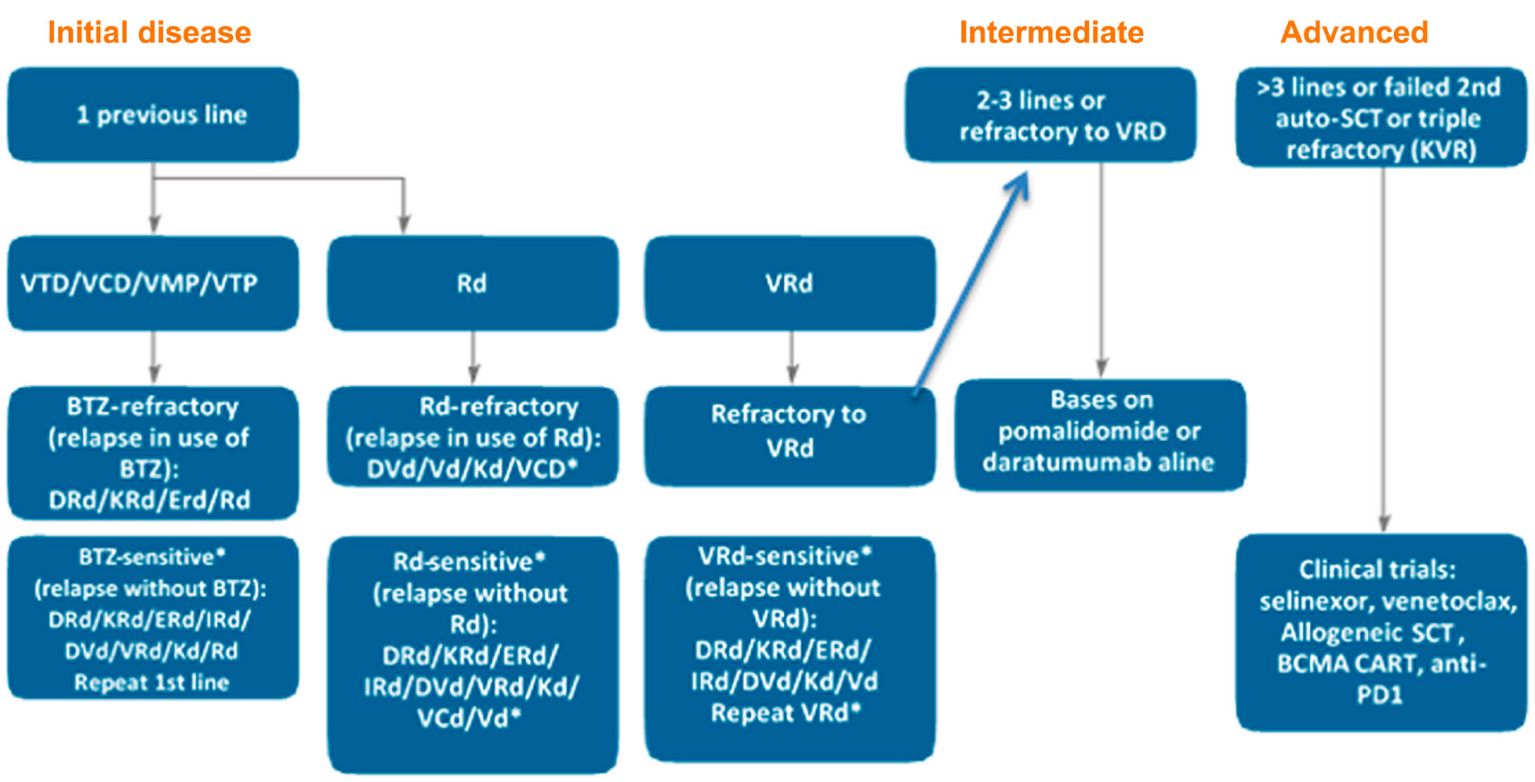
Proposed algorithm to treat early, intermediate and advanced disease (based on the number of previous lines). ^*^In Brazil, pomalidomide is not available (PCd, KPd, DPd, VPd and Pd are alternative options). IRd and ERd have a significant effect on PFS in patients with second and third previous lines not refractory to lenalidomide and preferably sensitive to bortezomib [13–15, 31, 86]. Patients who relapse after ≥ 2 years are considered sensitive.

The treatment suggested for RRMM is described in Figure 5 and is based on type and sensitivity to the previous therapy, duration of response and available drugs (Supplementary Table 3). DRd and KRd improved PFS in patients with 1 or 2–3 previous lines - the same for DVd and Kd. For IRd and ERd, the greatest benefit in PFS occurred in patients with 2–3 previous lines. In real life, 61% start a second line (10% disease progression, 4% patient refusal and 1% toxicity), 38% start a third line (16% disease progression, 6% refusal and 4% toxicity) and only 15% start a fourth line (24% by disease progression, 8% refusal and 2% toxicity) [64].

### Lifestyle: patient preferences, hospital visits, travel, infusion route and time

Suboptimal adherence or treatment interruption by the patient may be related to the low understanding or acceptance of treatment by the patient and have a negative impact on survival.

A strategy to humanize the medical prescription in our public service and make it clear to illiterate or difficult-to-understand patients (mostly due to polypharmacy), is to place a colored sticker on each oral medication box or bottle, each color corresponding to a different drug. In parallel with the medical prescription, a drawing of the sun (which represents the morning), a plate with cutlery (which represents lunch) and a moon (which represents the night) is made, so that each colored ball (drug) is glued respectively in period that it must be taken.

None of the previous studies (Table 1) addressed patient preference with RRMM. Preferences may vary due to treatment availability, previous treatments, disease duration, lack of motivation due to treatment failures, AEs, and age. When considering rescue treatment, it is essential to review the patient’s preferences and lifestyle [7, 65, 66].

A study about German RRMM patients preferences with novel proteasome inhibitor-based combination treatments showed patients were more interested in the application mode of the therapy, followed by higher efficacy compared with safety. “Therapy application regimen” was assigned the highest importance for treatment decisions (38.8%), followed by “time without progression of disease” (38.7%), “possibility of AE heart failure” (13.9%) and “possibility of AEs affecting the blood” (8.6%). Patients preferred oral intake once a day and once a week over other application modes and the highest overall utility was derived for IRd (utility: 3.218), compared with Rd (2.769), and KRd (1.928) [67].

A study used TSQM-9 (a general measure of patients’ satisfaction with medication) to investigate satisfaction with current treatment in patients with NDMM and showed that oral administration route of current therapy was a predictor of higher patient-perceived convenience while the use of therapies containing an injectable agent was associated with increased activity impairment (more work time missed and impairment while working), increased time burden, and higher indirect costs ($US482 versus 153) of MM therapy for patients and caregivers compared with solely orally administered therapies [68]. Similar data were observed for RRMM patients. ‘Chari et al. Tx Satisfaction in RRMM. The Oncologist. 2019. Another study reported that patients with MM using an oral-only regimen reported fewer clinic visits in the past 3 months, lower out-of-pocket costs for these visits, and less time spent at appointments related to MM treatments in the past month than did those receiving an injectable regimen [69].

Oral drugs may be preferable for patients who want to travel or live far from the reference center, as they reduce visits to the clinic. For example, the number of visits in 18 cycles is 18 for Rd, 18 for IRd, 28 for DRd, 96 for KRd (biweekly) and 48 for KRd (weekly) [15, 25, 26, 70].

Some patients refuse lenalidomide, fearing a second primary neoplasm (Table 3) [11, 12, 17, 25], and choose a non-lenalidomide regimen (DVd or Kd) instead. In our experience, others accept treatment but skip doses to be less exposed. Some prefer not to apply for health insurance coverage of lenalidomide (not yet on the list) and opt for DVd or Kd.

Continuous oral thalidomide is rarely used for RRMM, as it is generally been used as only one component of a multidrug first-line treatment (cyclophosphamide, thalidomide and dexamethasone; bortezomib, thalidomide and dexamethasone; melphalan, prednisone and thalidomide) and has specific intolerable AEs (PN, constipation or bradycardia) in some cases [48, 49, 52, 54].

Lenalidomide (5 mg, 10 mg, 15 mg and 25 mg capsules) may be used in regimens ranging from 14 to 21 days or continuously; taken at the same time, it contains lactose and, due to thrombotic risk, should be associated with acetylsalicylic acid or anticoagulants (if high risk) [40].

Pomalidomide (1 mg, 2 mg, 3 mg and 4 mg capsules), another immunomodulatory and antiangiogenic drug, is lactose free and is taken orally at 4 mg/day on days 1–14 (of a 21-day cycle) or 1–21 (of a 28-day cycle). The most common grade 3–4 adverse event associated with pomalidomide plus low-dose dexamethasone is myelosuppression. Aspirin prophylaxis is generally recommended for patients with a standard risk of venous thromboembolism, and low-molecular-weight heparin (prophylactic dose) or vitamin K antagonists (international normalized ratio 2–3) are recommended for patients with a high risk of venous thromboembolism [24, 43].

Ixazomib, the first oral proteasome inhibitor (2.3 mg, 3 mg or 4 mg capsule), has a weekly dosage (D1, D8 and D15), with rest in the last week of the 28-day cycle; this drug, taken at least one hour before or two hours after food, does not contain lactose. Antivirals should be considered for herpes zoster prevention [41].

Currently, bortezomib is preferentially given subcutaneously once a week, which causes fewer AEs than biweekly intravenous administration, reducing patient time in the hospital [57, 58]. Bortezomib may produce allergic reactions at the application site but is usually well tolerated [42]. Intravenous carfilzomib, 60 mg/vial, was previously applied twice a week (a reason for refusal by several patients), implying two weekly hospital visits for two consecutive days—a disadvantage compared to daratumumab which is administered weekly. Recently, the A.R.R.O.W. study [70] demonstrated similar results between biweekly and weekly dosages (with the dose increased to 70 mg/m^2^) for the Kd regimen, with fewer hospital visits positively impacting quality of life. Some patients using carfilzomib due to infusion-associated phlebitis require a long-term venous catheter (port-a-cath or peripherally inserted central venous catheter), a factor that may also impact treatment choice and quality of life. Doses of 20/27 mg/m^2^ are given within 10 minutes and 20/56/70 mg/m^2^ within 30 minutes, an advantage over daratumumab. The median PFS was increased in the weekly Kd group (11.2 months versus 7.6 months, *p* = 0.0029), and the incidence of grade ≥ 3 AEs was also slightly increased (68% versus 62%). A smaller proportion of patients had grade ≥ 3 congestive heart failure in weekly versus biweekly Kd (3% versus 4%) [70].

Regarding daratumumab, in addition to premedication, the infusion takes longer due to the incidence and seriousness of related infusion reactions with infusion times for the first, second and subsequent infusions of 6.5, 4.5 and 3.5 hours, respectively [71]. The addition of 10 mg of montelukast as a premedication before the first daratumumab infusion reduced one-third of the related infusion reactions [72]. Overall, 48% had related infusion reactions, 92% (cough, dyspnea, vomiting) at first infusion (5.3% G3), and one patient discontinued [23]. A study showed that increasing the infusion rate as of the third daratumumab infusion did not affect the safety profile [72]. The 90-minute infusion of daratumumab was well tolerated, and with this, patients gained two hours a day [72]. Subcutaneous formulation was approved for FDA recently based on findings from the phase 3 COLUMBA (MMY3012) study (COLUMBA; NCT03277105).

Elotuzumab (300 mg and 400 mg vial) was started at the infusion rate of 0.5 mL/min and progressively increased up to 5 mL/min after 3–4 cycles (10 mg/kg, ~3 h infusion). There are 10% related infusion reactions, with 70% in the first dose and 1% discontinuity [73].

Panobinostat (10 mg, 15 mg and 20 mg capsules) is a potent oral pan-deacetylase inhibitor for RRMM that has received between one and three previous treatment regimens. It is taken at a dose of 20 mg once every other day for 3 doses each week during weeks 1 and 2 of a 21-day treatment cycle. Determine the Fridericia-corrected QT interval prior to the start of therapy and verify that it is < 450 msec prior to panobinostat initiation once severe and fatal cardiac ischemic events, severe arrhythmias, and electrocardiogram changes have occurred in patients receiving panobinostat. Common grade 3–4 included thrombocytopenia (256 [67%] in the panobinostat group vs 118 [31%] in the placebo group), diarrhea (97 [26%] vs 30 [8%]), and PN (67 [18%] vs 55 [15%]) [22].

Selinexor (20 mg tablet) is an oral selective inhibitor of nuclear export compound that blocks exportin 1 and leads to activation of tumor suppressor proteins and inhibition of nuclear factor κB with activity in triple-class refractory MM compounds. This drug is taken at 80 mg/dose twice weekly on days 1 and 3 each week. Grade ≥ 3 common adverse reactions reported in at least 20% of patients included thrombocytopenia, anemia, neutropenia and hyponatremia [74].

Thus, DRd, KRd and ERd require venous access and prolonged hospital visits. On the other hand, oral drugs require the patient to remember to take the medication and use it on appropriate days, requiring the patient and family to have at least a minimum understanding of the dosage. Nausea and vomiting may further compromise efficacy and should be managed. In patients who cannot ingest drugs orally, these medications cannot be crushed or administered by probe, and subcutaneous or intravenous medication should be chosen.

In addition, drug doses should be adapted based on patient characteristics to avoid excessive toxicities leading to treatment discontinuation, negatively affecting survival or quality of life. Lenalidomide, pomalidomide and ixazomib adjust for kidney function; pomalidomide, ixazomib, bortezomib and carfilzomib adjust for liver function [44].

### Coronavirus disease (COVID-19) outbreak

On April 26, 2020, the pandemic of the new coronavirus (SARS-CoV2 / COVID-19), which appeared in December 2019 in the city of Wuhan, China, had already affected 213 countries, with 2,883,603 confirmed cases and 198,842 confirmed deaths in the world (WHO, 2020). There are concerns that the COVID-19 could overwhelm health-care systems worldwide, as severe and critical disease were reported, respectively, in 14 and 5 percent of patients, since they require intensive care assistance [75]. Most of the fatal cases occurred in patients with advanced age or underlying medical comorbidities [75, 76]. China reported a case-fatality rate of 14.8% in patients aged ≥ 80 years (208 of 1408) and 8.0% in patients aged 70–79 years (312 of 3918) [75]. Italian data reported that 20% of those who died from COVID-19 in the country had active cancer [77]. Patients with MM could be at particular risk from COVID-19, since they tend to be older, have multiple comorbidities as previously discussed, and they are immunosuppressed by their disease or therapy.

In such situation, caregivers should minimize their patients’ exposure to health-care facilities and many groups are issuing guidance. ESMO recommended oncologists to adjust their routines and suggested bolstering telemedicine services, reducing clinic visits, and switching to subcutaneous or oral therapies, rather than intravenous ones, whenever possible [78]. EBMT recommended that non-urgent transplants should be deferred as much as possible [79].

For patients with MM, treatment can be individualized to limit additional exposure to COVID-19. According to the American Society of Hematology, a reasonable approach is starting triplet therapy with VRd for 6–12 cycles followed by lenalidomide maintenance in patients requiring treatment, and bortezomib can be added to this every 2 weeks for high-risk patients. Postponing the stem cell transplant (including hematopoietic stem and progenitor cells collection and storage) is recommended [80]. Rd is a rational choice for frail patients or after achieving best response. Maintenance therapy should go on, since the risk of myeloma relapse is higher without treatment, but if a patient gets COVID-19, treatment should be interrupted until infection resolution. In order to decrease clinic visits, strategies such as telemedicine check-ins, in-home blood draws as required and prescription delivery via mail should be advised [80].

A recent publication of Sorbonne University recommended during pandemic of COVID-19 to complete six cycles of induction regimens in all patients with MM to postpone the transplant procedure. Additional cycles of induction until first relapse should be considered in standard-risk MM; however, for high-risk cytogenetics (especially those with deletion of chromosome 17p) it was recommended to follow with auto-SCT as first-line treatments whenever possible. They encouraged reduction (as for elderly patients) or interruption of steroids in patients in complete remission, change the treatment administration schedule to one with a lower frequency, change daratumumab to every 4 weeks instead of every 2 weeks after the initial 8-week weekly administration, in patients with very good partial response; and switch from an intravenous or subcutaneous treatment to a fully oral treatment combination [81].

For bisphosphonate intravenous home administration, switch from an intravenous to an oral bisphosphonate, switch to zometa every 3 months or transient interruption are supported [80].

For RRMM patients oral salvage chemotherapy with IRd may be a reasonable option to reduce COVID-19 exposure.

## CONCLUSIONS

Of the ten new antimyeloma drugs, seven are oral, simplifying intake and increasing patients’ autonomy and quality of life, but these drugs also reduce the number of hospital visits and transfer the responsibility of treatment management to the patient. The best treatment may not work if the patient is unable to accomplish it, whether due to forgetfulness, AEs or lack of social support. Completely oral rescue protocols such as IRd are options for patients who do are unwilling or unable to go to the hospital every week, providing the benefits of triple therapies (including proteasome inhibitors and immunomodulatory drugs) against progression without increasing the burden on hospital resources. Treatment with oral chemotherapy is of special value during the COVID-19 outbreak. In clinical practice, choosing the optimal combination for each patient is a challenge. Relapse treatment is influenced by previous treatment lines, efficacy and safety, convenience, quality of life, preferences, and personal feelings, among others, and should be considered and discussed with each patient. It is essential to individualize the approach of elderly MM patients, recognizing logistical, perceptual, physiological and socioeconomic barriers that compromise adherence and, thus, treatment efficiency. “Intake reminder methods” such as alarms, diaries, applications, and “pill boxes” are effective; however, patient education and communication with healthcare professionals are critical for adherence.

## SUPPLEMENTARY MATERIALS









## Abbreviations

AEs: adverse events
auto-SCT: autologous hematopoietic stem cell transplantation
PVd: pomalidomide, bortezomib, and dexamethasone
CrCl: creatinine clearance
COPD: chronic obstructive pulmonary disease
DRd: daratumumab, lenalidomide, and dexamethasone
DVd: daratumumab, bortezomib, and dexamethasone
ECOG: Eastern Cooperative Oncology Group performance status
ERd: elotuzumab, lenalidomide, and dexamethasone
IMWG: The International Myeloma Working Group
IRd: ixazomib, lenalidomide, and dexamethasone
Kd: carfilzomib and dexamethasone
KRd: carfilzomib, lenalidomide, and dexamethasone
MM: Multiple myeloma
NDMM: newly diagnosed multiple myeloma
RRMM: relapsed and/or refractory multiple myeloma
OS: overall survival
PFS: progression-free survival
PN: peripheral neuropathy
Pan-Vd: panobinostat, bortezomib, and dexamethasone
Rd: lenalidomide and dexamethasone
Vd: bortezomib and dexamethasone
VMP: bortezomib, melphalan and prednisone
