# Supplementary material for: Treatment of elderly patients with refractory/relapsed multiple myeloma: oral drugs adherence and the COVID-19 outbreak
Source: Oncotarget. 2020 Nov 24;11(47):4371–86. doi: 10.18632/oncotarget.27819 (PMC7720774; doi:10.18632/oncotarget.27819)
Supplement: Supplementary file 1 [file oncotarget-11-4371-s001.pdf]

Treatment of elderly patients with refractory/relapsed multiple myeloma: oral drugs adherence and the COVID-19 outbreak

SUPPLEMENTARY MATERIALS

Supplementary Table 1: Age and ECOG in the rescue treatment. See Supplementary Table 1

Supplementary Table 2: High-risk and rescue cytogenetics. See Supplementary Table 2

Supplementary Table 3: Previous lines and sensitivity to the treatment (PFS). See Supplementary Table 3

SUPPLEMENTARY REFERENCES

1. Palumbo A, Stewart AK, Rajkumar SV, Masszi T, Spicka I, Oriol A, Hájek R, Rosiñol L, Siegel D, Mihaylov GG, Goranova-Marinova V, Rajnics P, Suvorov A, et al. Efficacy and Safety of Carfilzomib, Lenalidomide, and Dexamethasone (KRd) vs Lenalidomide and Dexamethasone (Rd) in Patients (Pts) With Relapsed Multiple Myeloma (RMM) Based on Age: Secondary Analysis From the Phase 3 Study ASPIRE (NCT01080391). Clin Lymphoma Myeloma Leuk. 2015; 15:e75–6. https://doi.org/10.1016/j.clml.2015.07.227.

2. Mateos MV, Masszi T, Grzasko N, Hansson M, Sandhu I, Pour L, Viterbo L, Jackson SR, Stoppa AM, Gimsing P, Hamadani M, Borsaru G, Berg D, et al. Impact of prior therapy on the efficacy and safety of oral ixazomib-lenalidomide-dexamethasone vs. placebo-lenalidomide-dexamethasone in patients with relapsed/refractory multiple myeloma in TOURMALINE-MM1. Haematologica. 2017; 102:1767–75. https://doi.org/10.3324/haematol.2017.170118. [PubMed]
