# Supplementary material for: Treatment of elderly patients with refractory/relapsed multiple myeloma: oral drugs adherence and the COVID-19 outbreak
Source: Oncotarget. 2020 Nov 24;11(47):4371–86. doi: 10.18632/oncotarget.27819 (PMC7720774; doi:10.18632/oncotarget.27819)
Supplement: Supplementary file 2 [file oncotarget-11-4371-s002.docx]

**Supplementary Table 1: Age and ECOG in the rescue treatment**

| ***Studies*** | ***POLLUX*** [11, 12, 17, 29] | ***ASPIRE*** [26–30] | ***ELOQUENT-2*** [13, 14, 31] | ***TOURMALINE–MM1*** [15] | ***CASTOR*** [11, 16–18] | ***ENDEAVOR*** [19, 20, 30] | **OPTIMISMM** [21] | **PANOMARA1** [22] |
| --- | --- | --- | --- | --- | --- | --- | --- | --- |
| Experimental versus Control | DRd versus Rd | KRd versus Rd | ERd versus Rd | IRd versus Rd | DVd versus Vd | Kd versus Vd | PVd versus Vd | Pan-Vd versus Vd |
| Number | 286 versus 283 | 396 versus 396 | 321 versus 325 | 360 versus 362 | 251 versus 247 | 464 versus 465 | 281 versus 278 | 387 versus 381 |
| Median age (years) | 65 versus 65 | 64 versus 65 | 67 versus 66 | 66 versus 66 | 64 versus 64 | 65 versus 65 | 67 versus 68 | 63 versus 63 |
| 65-74 years (%) | 43 versus 38 | 65-70: 21 versus 24 | 37 versus 38 | 40 versus 34 | 38 versus 35 | 35 versus 41 | 40 versus 40^#^ | 33 versus 35 |
| ≥ 75 years (%) | 10 versus 12 | ≥ 70 years: 6 versus 29 | 21 versus 19 | 13 versus 17 | 9 versus 14 | 17 versus 14 | 16 versus 17^$^ | 9 versus 7 |
|  |  | ≥ 75 years: 11 versus 13 |  |  |  |  |  |  |
| PFS 65-74 years HR | **0.4*** | < 70 years: **0.70*** (0.56-0.88) NR | ≥ 65 years: **0.75*** (0.59-0.95) | 0.83 | ≥ 65 years: **0.35*** | **0.53*** (0.38-0.73) NR | NR | **≥ 65 years: HR 0.72 (0.53-0.96)*** |
| **CI (%)** | (0.24-0.67) NR | 28.6 versus 17.6 | 20 versus 18 NR | NR NR | (0.22-0.57) NR | NR |  |  |
| median (months) | NA versus NA |  |  | 17.5 versus 17.6 | NA versus 6.7 |  |  |  |
| PFS ≥ 75 years HR | **0.11*** (0.02-0.51) NR | ≥ 70 years: | **0.63*** | 0.87 | ≥ 65 years: **0.35*** | **0.38*** (0.23-0.65) NR | NR | NR |
| **CI (%)** | NA versus 11.4 | 0.75 | (0.41-0.96) | NR NR | (0.22-0.57) NR | NR |  |  |
| median (months) |  | (0.53-1.08) NR | 9 versus 6 NR | 18.5 versus 13.1 | NA versus 6.7 |  |  |  |
|  |  | 23.8 versus 16 |  |  |  |  |  |  |
|  |  | ≥ 75 years: |  |  |  |  |  |  |
|  |  | 0.62 |  |  |  |  |  |  |
|  |  | (0.36-1.08) NR |  |  |  |  |  |  |
|  |  | 30 versus 17 |  |  |  |  |  |  |
| OS 65-74 years HR | NR | NR | ≥ 65 years: | NR | NR | 0.71* (0.51-0.98) NR | NR | NR |
| CI |  |  | 0.78 |  |  | NR |  |  |
| (%) |  |  | (0.58-1.03) |  |  |  |  |  |
| median (months) |  |  | 31 versus 25 |  |  |  |  |  |
|  |  |  | (3 years) NR |  |  |  |  |  |
| OS ≥ 75 years HR | NR | NR | 0.51* | NR | NR | 0.84 | NR | NR |
| CI (%) |  |  | (0.31-0.85) |  |  | (0.52-1.36) NR |  |  |
| median (months) |  |  | 13 versus 7 |  |  | NR |  |  |
|  |  |  | (3 years) NR |  |  |  |  |  |
| **ECOG (%) 0** | 48.6 versus 53 | 89.9 versus | 91 (0-1) | 51 versus 47 | NR | 48 versus 50 | 53 versus 49 | 45 versus 43 |
| **1** | 47.6 versus | 91.2(0-1)** | 9 (2) | 44 versus 46 |  | 45 versus 44 | 43 versus 43 | 49 versus 49 |
| **2** | 41.7 | 10.1 versus |  | 5 versus 7 |  | 7 versus 6 | 4 versus 8 | 5 versus 8 |
|  | 3.8 versus 5.3 | 8.8 (2) |  |  |  |  |  |  |

* Bold text indicates the most significant data (horizontal line of forest chart does not cross main vertical line or relative risk 1). **(s)** = p <0.05. **PFS** = progression-free survival. **OS** = overall survival. **ECOG** = Eastern Cooperative Oncology Group performance status. **HR** = hazard ratio. **CI** = confidence interval.
