# Supplementary material for: Treatment of elderly patients with refractory/relapsed multiple myeloma: oral drugs adherence and the COVID-19 outbreak
Source: Oncotarget. 2020 Nov 24;11(47):4371–86. doi: 10.18632/oncotarget.27819 (PMC7720774; doi:10.18632/oncotarget.27819)
Supplement: Supplementary file 3 [file oncotarget-11-4371-s003.docx]

**Supplementary Table 2: High-risk and rescue cytogenetics**

| **Column A** | **Column B** | **Column C** | **Column D** |  | | | | |
| --- | --- | --- | --- | --- | --- | --- | --- | --- |
| Question: Regarding PFS, could the experimental treatment… | …outperform the HR-C arm? | ... equal or outperform SR-C? | ... equal or outperform SR-Ex? |  |  |  |  |  |
| HR-Ex | HR-Ex less HR-C (months)* | HR-Ex less SR-C (months)* | HR-Ex less SR-Ex (months)* | *Cutoff* del17p | *Cutoff* t (4:14) | Del17p (%) | t (4;14) (%) | CA (%) |
| DRd [11, 12, 23, 25] | > ↑13 | ↑4.1 | ↓> 2.6 (NA) | > 50% | NR | 11 versus 10 | 4 versus 7 | 15 versus 17 |
| KRd [26–30] | ↑9 (23.1 versus 13.9, HR:0,7, IC: 0.43-1.16) | ↑3.6 23.1 versus 19.5) | ↓6.5 (23.1 versus 29.6) | ≥ 60% | NR | 9 versus 9 | 17 versus 14 | 25 versus 23 |
| ERd [13, 14] | ↑7.8** | ↓1.4** | ↓4.5** | ≥1% | NR | 32 versus 32 | 9 versus 10 | 19 versus 20** |
| IRd [15] | ↑11.7 (21.4 versus 9.7, HR 0.5, p = 0,021) | ↑5.8 (21.4-15.6) | ↑0.8 (21.4-20.6) | ≥ 5% | ≥ 3% | 10 versus 9 | 10 versus 7 | 21 versus 17 |
| BPd [21] | ↑3.12 (8.44 versus 5.32, HR 0.56, p=0.021) | NR | NR | NR | NR | NR | NR | 22 versus 18 |
| Pan-Vd [22] | NR | NR | NR | NR | NR | NR | NR | NR |
| DVd [11, 16–18]^1^ | ↑4 | ↑4.2 | ↓8.4 | > 50% | NR | NR | NR | 26 versus 27 |
| Kd [19, 20, 30] | ↑2.2 | ↓1.4 | ↓> 15.2 | ≥ 20% | ≥ 10% | 11 versus 13 | 13 versus 15 | 21 versus 24 |

**SR-Ex** = standard risk treated in the experimental arm. **SR-C** = standard risk treated in the control arm. **HR-Ex** = high risk treated in the experimental arm. **HR-C** = high risk treated in the control arm. **PFS** = progression-free survival. ***** = Difference in PFS medians. ****** = in ELOQUENT-2, the high-risk group was defined as ISSII/III + t (4;14) or del17p. ↑: increase. ↓: reduction.
