# Supplementary material for: Treatment of elderly patients with refractory/relapsed multiple myeloma: oral drugs adherence and the COVID-19 outbreak
Source: Oncotarget. 2020 Nov 24;11(47):4371–86. doi: 10.18632/oncotarget.27819 (PMC7720774; doi:10.18632/oncotarget.27819)
Supplement: Supplementary file 4 [file oncotarget-11-4371-s004.docx]

**Supplementary Table 3: Previous lines and sensitivity to the treatment (PFS)**

| ***Studies*** | ***POLLUX*** [11, 12, 17, 29] | ***ASPIRE*** [26–30, 1] | ***ELOQUENT-2*** [13, 14, 31] | ***TOURMALINE–MM1*** [15, 2] | ***CASTOR*** [11, 16–18] | ***ENDEAVOR*** [19, 20, 30] | **OPTIMISMM** [21] | **PANOMARA1** [22] |
| --- | --- | --- | --- | --- | --- | --- | --- | --- |
| Experimental versus Control | DRd versus Rd | KRd versus Rd | ERd versus Rd | IRd versus Rd | DVd versus Vd | Kd versus Vd | PVd versus Vd | Pan-Vd versus Vd |
| 1 previous line | **NA versus 20(s)* (HR: 0.39)** | **30 versus 18(s)* (HR: 0.69)** | HR: 0.77 (CI: 0.59-1.01) | 21 versus 17 (HR: 0.88. ns) | **NA versus 8 (HR: 0.19. CI: 0.12-0.29)*** | HR 0.45 (CI: 0.33-0.61)* | **HR 0.54 (CI: 0.36-0.82)(s)** | **NR** |
| 2-3 previous lines | **29 versus 16(s) (HR: 0.38)** | **26 versus 17(s) (HR: 0.68)** | **HR: 0.68 (CI: 0.53-0.87)*** | **NA versus 13 (HR: 0.5. CI: 0.4-0.8. p=0.0033)** | **10 versus 6 (HR: 0.51. CI: 0.36-0.71)** | HR 0.6 (CI: 0.47-0.78) | HR 0.6 (0.38-0.95)(s) | **NR** |
| Previous lenalidomide | **NA versus 19(s)** | HR 0.8 (CI: 0.52-1.22) | HR: 0.55 (CI: 0.24-1.25) | **IMiD: NA versus 18 (HR: 0.74. s) (12% lenalidomide/ 44% thalidomide)** | **10 versus 6 (HR: 0.38. CI: 0.26-0.56)** | HR: 0.69 (CI: 0.52-0.92) | 100% | IMiD: **HR 0.54 (0.43-0.68)** |
| Previous thalidomide | **NA versus 13(s)** | NR | HR: 0.68 (CI: 0.52-0.9) |  | **17 versus 7 (HR: 0.28. CI: 0.19-0.41)** | HR: 0.54 (CI: 0.41-0.71) | NR | NR |
| Previous bortezomib, previous PI | **NA versus 18(s)** | **HR 0.7 (CI: 0.56-0.88)** | **HR 0.66 (CI: 0.53-0.83)** | **18 versus 14 (HR: 0.74. s)** | **12 versus 7 (HR: 0.35. CI: 0.26-0.46)** | **HR 0.56 (CI: 0.44-0.73)** | **HR 0.57 (0.44-0.73)** | **HR: 0.58 (0.44-0.77)** |
| Refractory to bortezomib | **26 versus 11(s)** | HR: 0.8 (CI: 0.49-1.3) | 21% refractory | Excluded | Excluded | HR 0.37 (CI: 0.13-1.08) (≤3%) | 9% versus 12% | NR |
| Refractory to lenalidomide | Excluded | **IMiD: HR 0.64 (CI: 0.44-0.91)** | NR | Excluded | **9 versus 4, HR 0.36 (CI: 0.21-0.63)** | **HR 0.8 (CI: 0.57-1.11)** | 70% **HR 0.65 (CI: 0.5-0.84)(s)** | **NR** |

(s) = significant (*p* <0.05). IMiD = immunomodulatory drugs; NR = not reported; NA = not reached; KVR = carfilzomib, bortezomib or lenalidomide.
